# Anomalous aortic origin of right coronary artery from left coronary cusp: a management conundrum: a case report

**DOI:** 10.1186/s13256-023-03921-1

**Published:** 2023-05-10

**Authors:** Pramukh Arun Kumar, Boskey Patel, Mahati Dasari, Pradnya Brijmohan Bhattad, Sushmita Prabhu, Michelle Hadley

**Affiliations:** 1 Department of Internal Medicine, Saint Vincent Hospital, 123 Summer Street, Worcester, MA 01608 USA; 2 Division of Cardiovascular Medicine, Lahey Hospital & Medical Center, Burlington, MA USA; 3 Division of Cardiovascular Medicine, Saint Vincent Hospital, Worcester, MA USA

**Keywords:** Chest pain, Acute coronary syndrome, Adult congenital heart disease, Anomalous coronary artery, Right coronary artery

## Abstract

**Background:**

Coronary artery anomalies are characterized by an abnormality in the course or origin of three main coronary arteries. There needs to be more scientific evidence to promptly treat coronary artery anomalies with poorly understood prognostic implications, especially anomalous aortic origin of the right coronary artery from the left coronary cusp.

**Case presentation:**

A 58-year-old Caucasian female presented multiple times over 6 months with atypical chest discomfort and palpitations. The treadmill exercise test demonstrated exercise-induced non-sustained ventricular tachycardia. A coronary angiogram revealed no obstructive coronary artery disease and an anomalous aortic origin of the right coronary artery from the left coronary cusp with an interarterial course. She was managed conservatively with medications, despite persistent recurrent symptoms.

**Conclusion:**

It is essential to identify subtle symptoms and insidious onset of anomalous aortic origin of the right coronary artery symptoms as seen in our patient, which can contribute to significant morbidity. There are discrepancies in existing guidelines between different cardiovascular societies in managing selected subgroups of patients with anomalous aortic origin of the right coronary artery who do not have high-risk features, but continue to remain symptomatic.

**Supplementary Information:**

The online version contains supplementary material available at 10.1186/s13256-023-03921-1.

## Background

Coronary artery anomalies (CAA) include a rare collection of conditions characterized by an abnormality in the course or origin of any of the three main epicardial coronary arteries (right coronary artery, left coronary artery, and left anterior descending artery). It is present in less than 1% of the general population [1]. Even with advanced cardiac imaging, diagnosis of CAA remains elusive [2]. Furthermore, there is scanty scientific evidence to promptly treat CAA, especially anomalous aortic origin of the right coronary artery (AAORCA) from the left coronary cusp [3]. Here, we present a rare case of AAORCA arising from the left coronary cusp with no high-risk features but persistent symptoms, posing a management conundrum.

## Case presentation

### Presentation

A 58-year-old Caucasian female presented to the emergency department with chest pain episodes that were brief, sharp, anterior, non-radiating, and intermittent, with no other associated symptoms. Vital signs were unremarkable, except for elevated blood pressure at 148/85 mm Hg. Basic labs, including cardiac biomarkers, were within normal limits. Chest X-ray showed no acute process, and an electrocardiogram (ECG) showed no findings of ischemia or arrhythmia. Her medical history was significant for type 2 diabetes mellitus, obesity, hypertension, and hyperlipidemia. Home medications included amlodipine, aspirin, atorvastatin, and lisinopril. She had a 40-pack-year smoking history and quit the year before. She denied alcohol or recreational drug use and had no family history of premature CAD or sudden cardiac death. She was diagnosed with reflux and discharged home. She presented again 5 months later with a 15-minute episode of dyspnea and dizziness. Her physical exam was entirely benign, and her workup was negative for the acute coronary syndrome (ACS).

One year later, she was back with persistent palpitations for 24 hours. She felt fatigued and endorsed vague chest discomfort, but no shortness of breath or lightheadedness. She was diagnosed with new-onset atrial fibrillation with a rapid ventricular response. Computerized tomography of the chest revealed no pulmonary embolism. She received intravenous diltiazem with improvement in her heart rate and was discharged home on apixaban and oral diltiazem, with instructions to follow up with cardiology outpatient. Before her cardiology appointment, she presented to the emergency department again with a fluttering sensation in her chest associated with lightheadedness but no pain. Once again, the ACS workup did not reveal any signs of ischemia, and she was discharged home. Two days later, she was seen in the cardiology clinic and scheduled for a 30-day event monitor to assess atrial fibrillation burden, an echocardiogram to rule out structural abnormalities, and an exercise nuclear stress test for ischemia.

### Workup

**Fig. 1:**
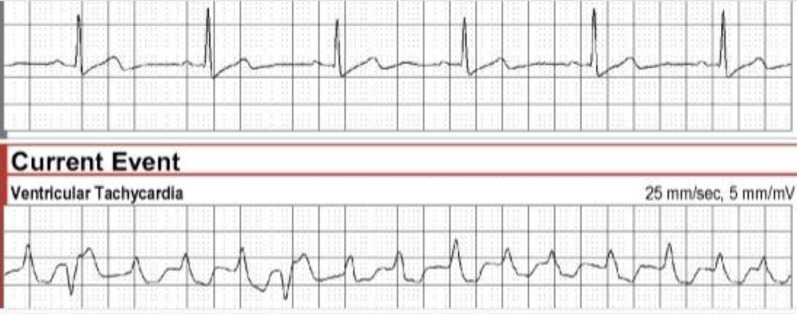
Event monitor tracing with baseline sinus rhythm (above) and a 17-beat run of ventricular tachycardia (below)

Initial ECG showed sinus rhythm, normal axis, and intervals, and no ischemic changes or arrhythmias. A transthoracic echocardiogram revealed normal left ventricle function and no wall motion abnormalities. The left ventricular ejection fraction was 68%. Myocardial perfusion imaging with stress-gated single photon emission computerized tomography (SPECT) and rest SPECT imaging was normal, without evidence of ischemia or infarction. Frequent premature ventricular contractions (PVCs), intermittent ventricular bigeminy, and short bursts of non-sustained ventricular tachycardia were noted at peak exercise and early recovery. A 30-day event monitor revealed sinus rhythm predominantly with an average heart rate of 62 beats per minute, although a few brief episodes of atrial fibrillation were noted. In addition, a 17-beat run of ventricular tachycardia (VT) at a rate of 173 beats per minute was detected on day 20 (Fig. 1). After these findings, her diltiazem was stopped in favor of metoprolol succinate.

### Diagnosis/management

**Fig. 2:**
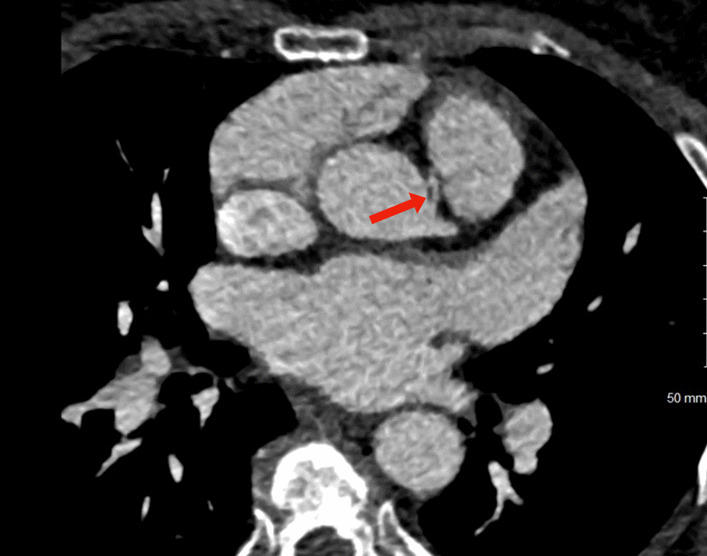
Anomalous right coronary artery (arrow) arising from the left coronary cusp

**Fig. 3:**
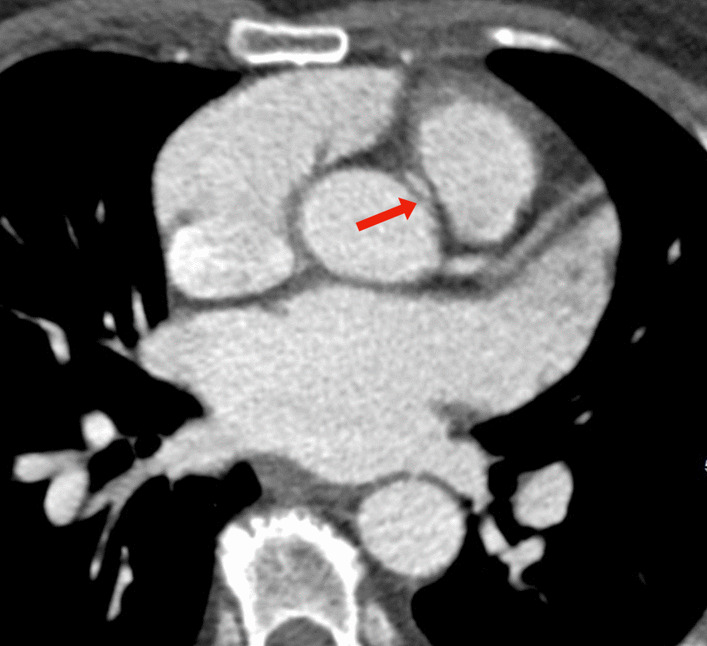
An interarterial course of anomalous right coronary artery (arrow) between ascending aorta and pulmonary trunk

Subsequent cardiac catheterization performed to assess for ischemic etiology of the exercise-induced non-sustained ventricular tachycardia showed no evidence of obstructive coronary disease. Interestingly, the dominant right coronary artery was noted to arise from the left coronary cusp with a shared ostium to the left system (Additional file 1: Video S1, Additional file 2: Video S2, Additional file 3: Video S3). In addition, a cardiac coronary computerized tomography scan confirmed the right dominant circulation with RCA arising from the left coronary cusp (Fig. 2) with an interarterial course (Fig. 3). Therefore, the patient was referred for a cardiothoracic surgery consultation. She continued to experience palpitations, lightheadedness, and occasional substernal pressure with exertion. However, in the absence of any signs of true myocardial ischemia (e.g., typical angina, ischemic findings on provocative testing, aborted sudden cardiac death or arrest, or non-vagally-mediated arrhythmia), and due to the lack of consensus regarding surgical treatment of AAORCA, a shared decision was made for conservative management per patient preference after multidisciplinary team discussion. The conservative management included restriction of vigorous exercise, up-titration of beta-blockers, and the addition of other anti-anginal agents. A loop recorder was also implanted for ventricular tachycardia (VT) monitoring.

### Follow-up

A few months after her consultation with the cardiothoracic surgeon, the patient was admitted to the hospital several times with a similar constellation of symptoms, including chest pain and left arm throbbing. Workup each time was unrevealing for an acute cardiac etiology. Her cardiac medications were optimized by up-titrating dosage of her beta blocker and adding isosorbide mononitrate as an additional antianginal agent, and her symptoms have since improved. Her implantable loop recorder showed one brief episode of atrial fibrillation with a rapid ventricular response, but no further episodes of VT.

## Discussion

The incidence of anomalous aortic origin of the right coronary arteries is low, with reported values between 0.026 and 0.25% [4]. Most AAORCA are asymptomatic, with a minority “malignant” with high-risk features, such as high interarterial, intramural course, high take-off, and a slit-like ostium [5]. Chest pain is the most common presenting symptom, followed by palpitations and syncope [6, 7]. Complications include sudden cardiac arrest or death, myocardial ischemia, and arrhythmias [8]. Although rare, complications commonly occur during or immediately after exercise [9]. Prognostic significance of AAORCA remains unclear, especially in non-athletes [3].

Although the surgical options for an anomalous interarterial left coronary artery have been well established by this point, it is controversial to surgically intervene for an AAORCA, particularly one arising from the left coronary cusp [10, 11]. A working group provided the first written consensus statement for practice guidelines on this topic. In this consensus statement, surgical intervention is recommended for those with signs or symptoms of myocardial ischemia (e.g., true angina, findings on provocative testing, aborted sudden cardiac death or arrest, or non-vagally-mediated arrhythmia) [12]. According to the 2018 ACC/AHA and 2020 ESC guidelines for managing adults with congenital heart diseases, surgery may be considered in our patient, but remains a class IIb recommendation with a low level of evidence [3].

Although our patient continued to experience chest pain and palpitations, her symptoms were atypical and did not meet the above-mentioned criteria that would prompt surgical intervention consideration. Fortunately, she has responded well to conservative management. However, in patients with limited response to medical management or deteriorating functional capacity, it would be reasonable to implement an individualized multidisciplinary team approach for each patient, including surgical intervention for improved quality of life.

## Conclusion

There is a rising incidence of AAORCA with the widespread use of cardiac CT scans and coronary angiograms. It is essential to identify subtle symptoms and insidious onset of AAORCA symptoms as seen in our patient, which can contribute to significant morbidity. A revision of guidelines for managing AAORCA from the left coronary cusp would be helpful to address such patients by utilizing a multidisciplinary team approach. Lastly, we need robust prospective studies to evaluate the benefits of surgical intervention compared with conservative management in a selected subgroup of patients with AAORCA who do not have high-risk features, but continue to remain symptomatic.

## Supplementary Information

**Additional file 1: Video S1.** RCA partially filling with contrast via the reflux from the left main injection.

**Additional file 2: Video S2.** Partial filling of the left system via reflux from injection of the RCA.

**Additional file 3: Video S3.** Catheter pullback demonstrates shared ostium between the RCA and the left coronary system.

## Data Availability

All data are included in the abstract.

## Abbreviations

AAORCA: Anomalous aortic origin of right coronary artery
ACS: Acute coronary syndrome
CAA: Coronary artery anomalies
CAD: Coronary artery disease
CT: Computerized tomography
ECG: Electrocardiogram
PVC: Premature ventricular contraction
SPECT: Single photon emission computerized tomography
VT: Ventricular tachycardia
